# Supplementary material for: P2X4 receptors mediate induction of antioxidants, fibrogenic cytokines and ECM transcripts; in presence of replicating HCV in in vitro setting: An insight into role of P2X4 in fibrosis
Source: PLoS One. 2022 May 20;17(5):e0259727. doi: 10.1371/journal.pone.0259727 (PMC9122194; doi:10.1371/journal.pone.0259727)
Supplement: S1 File — (1.7-kb) (Rattus norvegicus), kindly provided by Dr. Ishtiaq Qadri with reported sequence (Sequence chromatogram not shown). (PDF) [file pone.0259727.s001.pdf]

Formatting options   Download

Nucleotide Sequence (3798 letters)

**Query ID**	lcl|52517	**Database Name**	gpipe/10116/all_contig
**Description**	None	**Description**	rat build 4 genome database alternate assemblies)
**Molecule type**	nucleic acid		
**Query Length**	3798	**Program**	BLASTN 2.2.22+ Citation

```
>ref|NW_047375.1|Rn12_WGA1896_4 D Rattus norvegicus chromosome 12 genomic contig, reference assembly
(based on RGSC v3.4)
Length=7447876

 Features in this part of subject sequence:
   purinergic receptor P2X4


 Score =  167 bits (90),  Expect = 9e-38
 Identities = 98/101 (97%), Gaps = 3/101 (2%)
 Strand=Plus/Minus

Query  1771     CCTGGATAGAGCCGCCTCCCTTTGCCTGCCCAGATATTCCTTCCGGCGCCTGGACACCCG  1830
                ||||||||||||||||||||||||||||||||||||||||||||||||||||||||||||
Sbjct  6381555  CCTGGATAGAGCCGCCTCCCTTTGCCTGCCCAGATATTCCTTCCGGCGCCTGGACACCCG  6381496

Query  1831     GGACCTG-AACACA-TGTGTCTCCTGGCTACA-TTTCAGGT  1868
                ||||||| |||||| ||||||||||||||||| ||||||||
Sbjct  6381495  GGACCTGGAACACAATGTGTCTCCTGGCTACAATTTCAGGT  6381455
```

```
 Features in this part of subject sequence:
   purinergic receptor P2X4


 Score =  102 bits (55),  Expect = 2e-18
 Identities = 55/55 (100%), Gaps = 0/55 (0%)
 Strand=Plus/Plus

Query  2966     CTATGTCCCGCGTCCTCCACGATTGTGCCAAGACGGAATATGGGGCAGAAGGGAT  3020
                |||||||||||||||||||||||||||||||||||||||||||||||||||||||
Sbjct  6381710  CTATGTCCCGCGTCCTCCACGATTGTGCCAAGACGGAATATGGGGCAGAAGGGAT  6381764


 Features in this part of subject sequence:
   purinergic receptor P2X4


 Score =  100 bits (54),  Expect = 9e-18
 Identities = 54/54 (100%), Gaps = 0/54 (0%)
 Strand=Plus/Minus

Query  523      ACCAGGAAACGGACTCCGTGGTCAGCTCGGTGACAACCAAAGCCAAAGGTGTGG  576
                ||||||||||||||||||||||||||||||||||||||||||||||||||||||
Sbjct  6389830  ACCAGGAAACGGACTCCGTGGTCAGCTCGGTGACAACCAAAGCCAAAGGTGTGG  6389777
```

Figure : Sequence homology of full length P2X4 sequence (1.7-kb) (*Rattus norvegicus*), kindly provided by Dr. Ishtiaq Qadri with reported sequence (Sequence chromatogram not shown
