# Supplementary material for: P2X4 receptors mediate induction of antioxidants, fibrogenic cytokines and ECM transcripts; in presence of replicating HCV in in vitro setting: An insight into role of P2X4 in fibrosis
Source: PLoS One. 2022 May 20;17(5):e0259727. doi: 10.1371/journal.pone.0259727 (PMC9122194; doi:10.1371/journal.pone.0259727)
Supplement: S2 File — (PDF) [file pone.0259727.s002.pdf]

File: 02-T7-~1.AB1 Sequence Name: 02-T7-SEP-48 Run ended: Mar 27, 2009

10 20 30 40 50 60 70

CNANGGCTNCGTTAACTTAGCTTGATATCGAATTCCTGCAGCCCGGGGGATCCGCCCACCCNCAGTGTCCAAGGCGCG

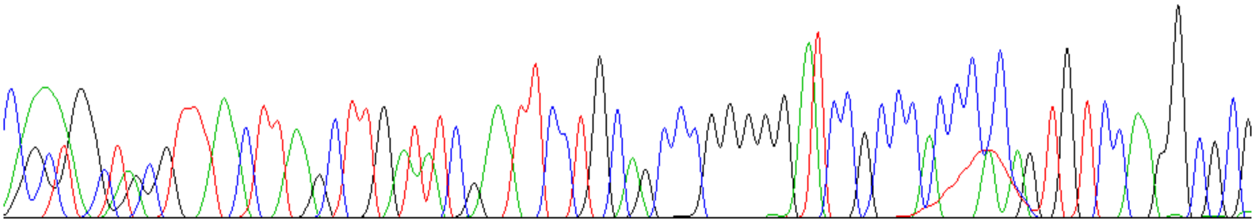

File: 02-T7-~1.AB1 Sequence Name: 02-T7-SEP-48 Run ended: Mar 27, 2009

80 90 100 110 120 130 140 150

GCGCGGAAGCGGTCGGCGGAGCCATGGCGGGCTGCTGCTCCGTGCTCGGGTCCTTCCTGTTCGAGTACGACACGCCG

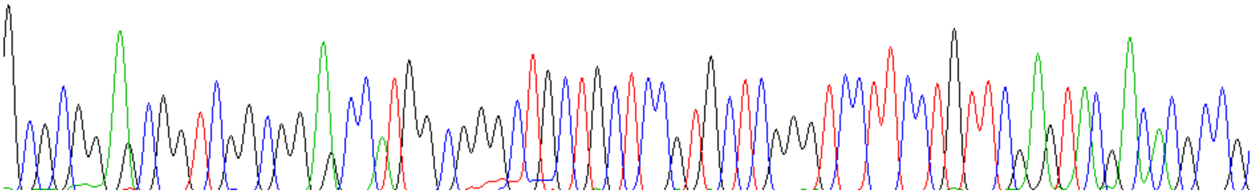

File: 02-T7-~1.AB1 Sequence Name: 02-T7-SEP-48 Run ended: Mar 27, 2009

160 170 180 190 200 210 220 230

CATCGTGCTCATCCGCAGCCGTAAAGTGGGGCTCATGAACCGCGCGGTGCAGCTGCTCATCCTGGCTTACGTCATCGG

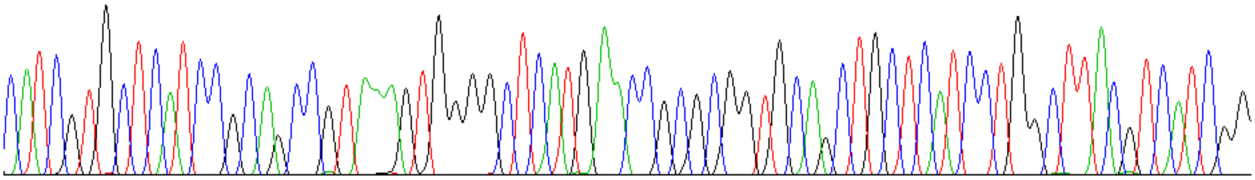

File: 02-T7-~1.AB1 Sequence Name: 02-T7-SEP-48 Run ended: Mar 27, 2009

240 250 260 270 280 290 300 310

GGGTGTTCGTGTGGGAAAAGGGCTACCAGGAAACGGACTCCGTGGTCAGCTCGGTGACAACCAAAGCCAAAGGTGTGGC

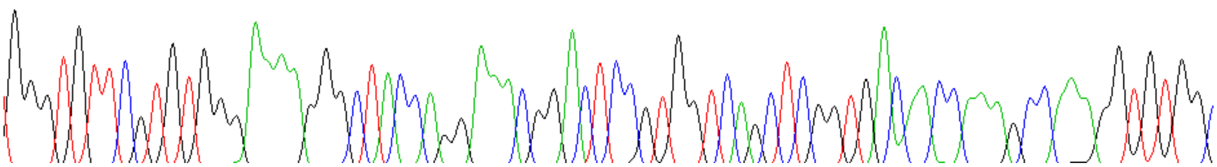

File: 02-T7-~1.AB1 Sequence Name: 02-T7-SEP-48 Run ended: Mar 27, 2009

0 320 330 340 350 360 370 380

CTGTGACCAACACCTCTCAGCTTGGATTCCGGATCTGGGACGTGGCGGACTATGTGATTCCAGCTCAGGAGGAAAACTC

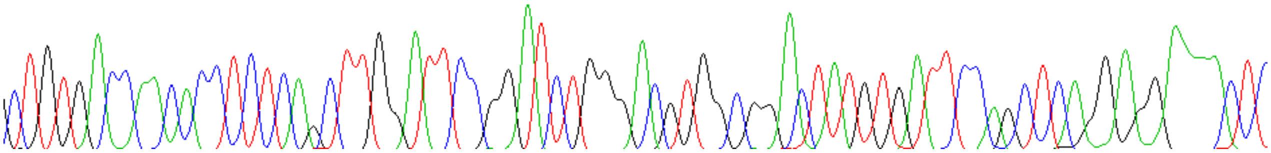

File: 02-T7-~1.AB1 Sequence Name: 02-T7-SEP-48 Run ended: Mar 27, 2009

390 400 410 420 430 440 450 460

AAACTCCCTCTTCATTATGACCAACATGATTGTCACCGTGAAACCAGACACAGAGCACCTGTCCAGAGATTCCTGATAAG

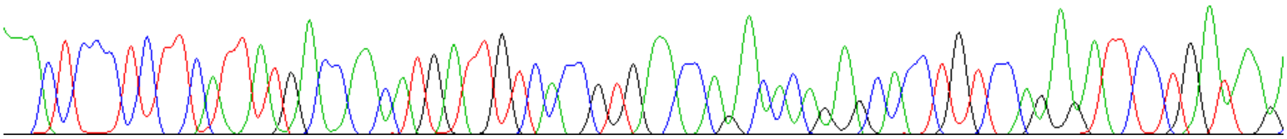

File: 02-T7-~1.AB1 Sequence Name: 02-T7-SEP-48 Run ended: Mar 27, 2009

470 480 490 500 510 520 530 540

ACCAGCATTTGTAATTCAGACGCCGACTGCACTCCTGGCTCCGTGGACACCCACAGCAGTGGAGTTGCGACTGGAAGATC

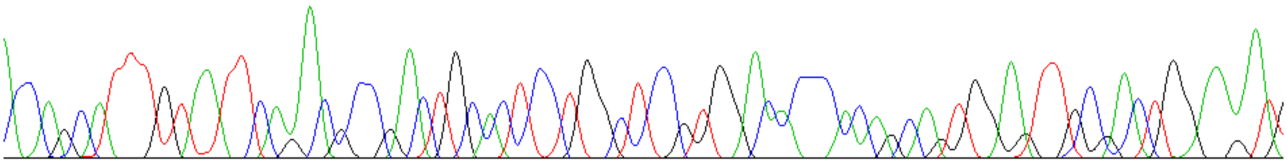

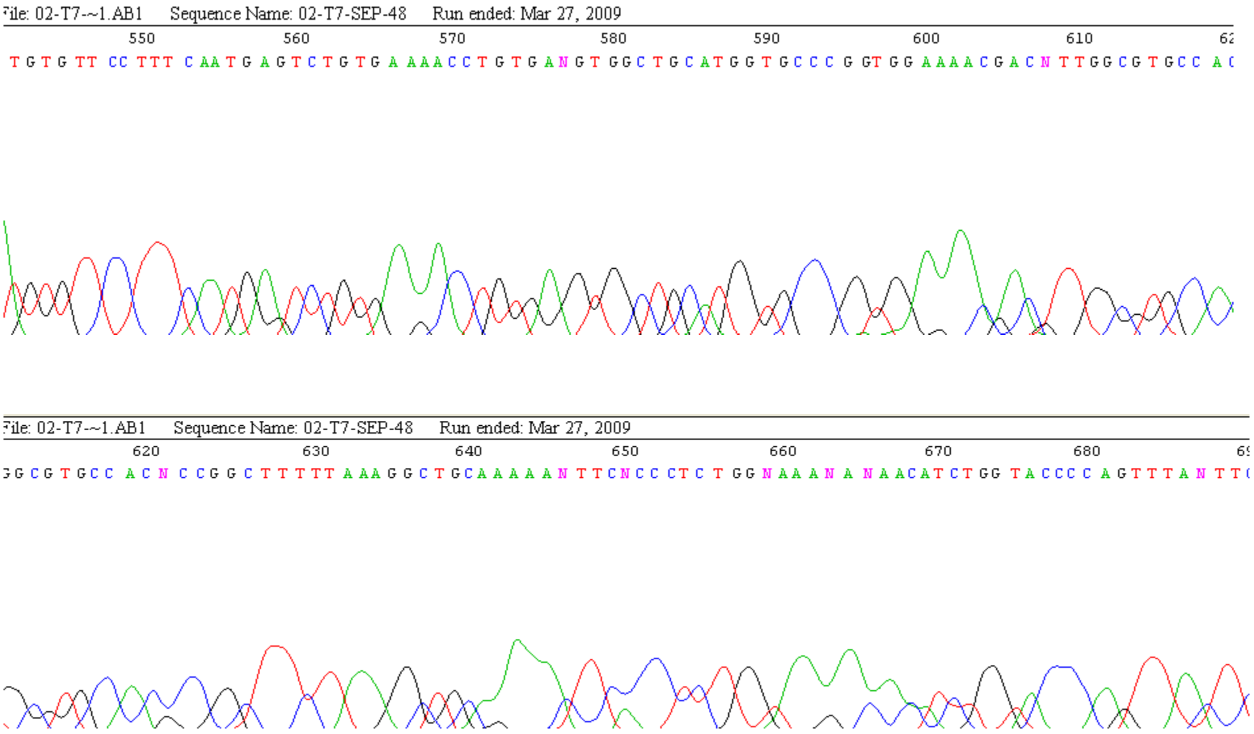

Figure : Representative chromatograms of sequence of P2X4 receptor (1.7-kb) before DNA transfection.

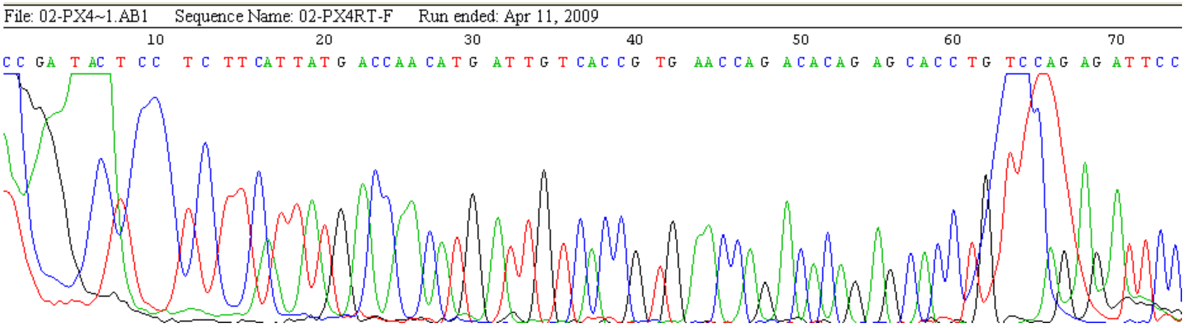

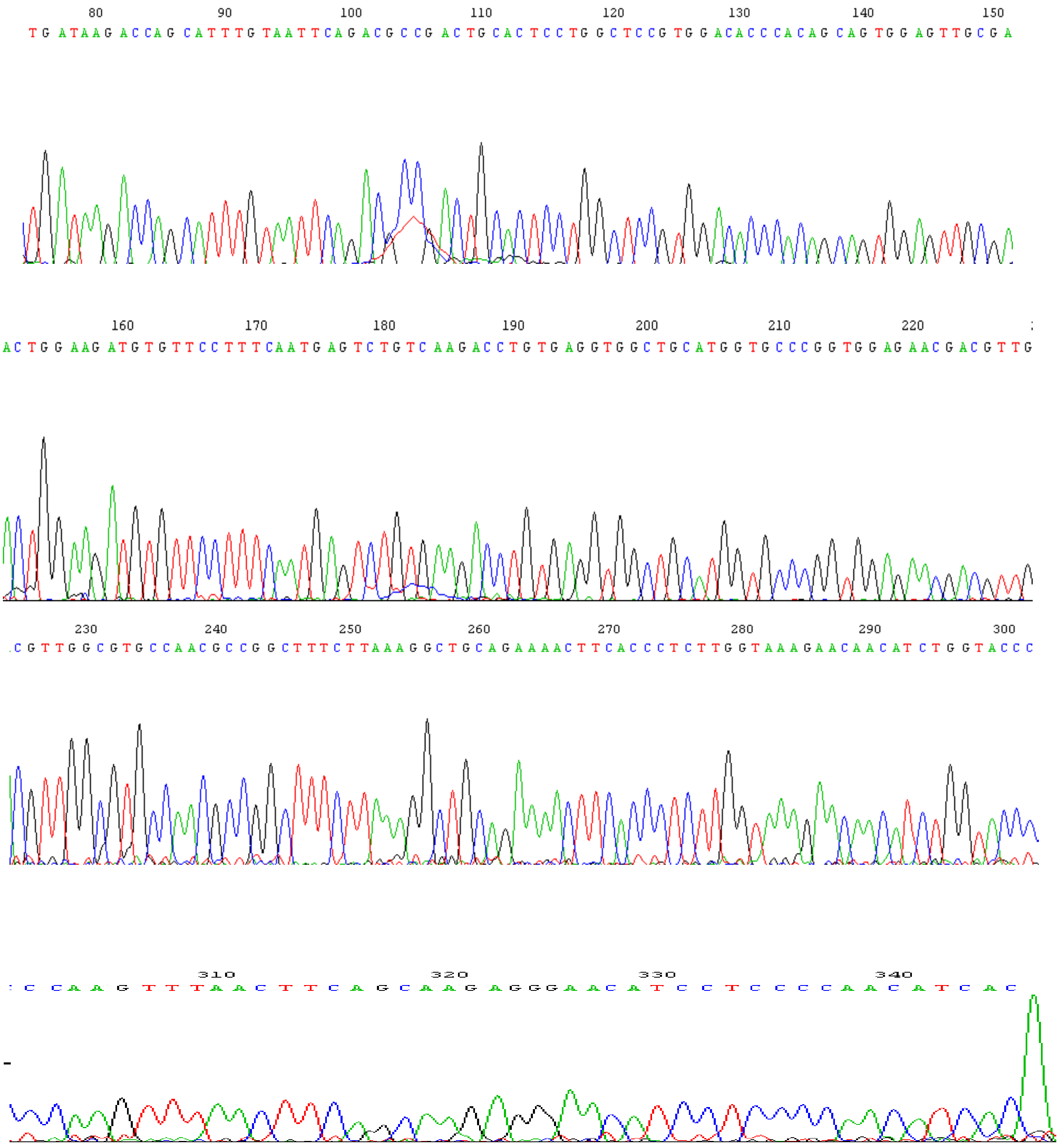

Figure : Representative chromatograms of sequence of transfected P2X4 receptor amplified from Stable cell line (293T/P2X4).
