# Supplementary material for: P2X4 receptors mediate induction of antioxidants, fibrogenic cytokines and ECM transcripts; in presence of replicating HCV in in vitro setting: An insight into role of P2X4 in fibrosis
Source: PLoS One. 2022 May 20;17(5):e0259727. doi: 10.1371/journal.pone.0259727 (PMC9122194; doi:10.1371/journal.pone.0259727)
Supplement: S3 File — Ishtiaq Qadri along with full length gene P2X4 (1.7-kb). (PDF) [file pone.0259727.s003.pdf]

Blast 2 sequences

Nucleotide Sequence (3798 letters)

**Query ID**	lcl|8471	**Subject ID**	8473
**Description**	None	**Description**	02-PX4~1 sequence exported from chromatogram file
**Molecule type**	nucleic acid	**Molecule type**	nucleic acid
**Query Length**	3798	**Subject Length**	696
		**Program**	BLASTN 2.2.22+ Citation

```
 Identities = 53/53 (100%), Gaps = 0/53 (0%)
 Strand=Plus/Minus

Query  3488  ACTCATTGAAAGGAACACATCTTCCAGTCGCAACTCCACTGCTGTGGGTGTCC  3540
             |||||||||||||||||||||||||||||||||||||||||||||||||||||
Sbjct  178   ACTCATTGAAAGGAACACATCTTCCAGTCGCAACTCCACTGCTGTGGGTGTCC  126


 Score = 99.0 bits (53),  Expect = 4e-24
 Identities = 53/53 (100%), Gaps = 0/53 (0%)
 Strand=Plus/Plus

Query  1146  CCTGTGAGGTGGCTGCATGGTGCCCGGTGGAGAACGACGTTGGCGTGCCAACG  1198
             |||||||||||||||||||||||||||||||||||||||||||||||||||||
Sbjct  188   CCTGTGAGGTGGCTGCATGGTGCCCGGTGGAGAACGACGTTGGCGTGCCAACG  240


 Score = 99.0 bits (53),  Expect = 4e-24
 Identities = 53/53 (100%), Gaps = 0/53 (0%)
 Strand=Plus/Minus

Query  1146  CCTGTGAGGTGGCTGCATGGTGCCCGGTGGAGAACGACGTTGGCGTGCCAACG  1198
             |||||||||||||||||||||||||||||||||||||||||||||||||||||
Sbjct  475   CCTGTGAGGTGGCTGCATGGTGCCCGGTGGAGAACGACGTTGGCGTGCCAACG  423


 Score = 99.0 bits (53),  Expect = 4e-24
 Identities = 53/53 (100%), Gaps = 0/53 (0%)
 Strand=Plus/Minus

Query  728   CTATGTGATTCCAGCTCAGGAGGAAAACTCCCTCTTCATTATGACCAACATGA  780
             |||||||||||||||||||||||||||||||||||||||||||||||||||||
Sbjct  684   CTATGTGATTCCAGCTCAGGAGGAAAACTCCCTCTTCATTATGACCAACATGA  632
```

Figure : Sequence homology of transfected P2X4 (stable cell line 293T/P2X4) with sequence kindly provided by Dr.Ishtiaq Qadri along with full length gene P2X4 (1.7-kb).
