# Supplementary material for: P2X4 receptors mediate induction of antioxidants, fibrogenic cytokines and ECM transcripts; in presence of replicating HCV in in vitro setting: An insight into role of P2X4 in fibrosis
Source: PLoS One. 2022 May 20;17(5):e0259727. doi: 10.1371/journal.pone.0259727 (PMC9122194; doi:10.1371/journal.pone.0259727)
Supplement: S4 File — 100X), 293T/NV cell line experimental and control in presence of G418(c, d. 100X). 293T/P2X4 cell line and 293T/NV cell line demonstrated better resistance to G418 in comparison to both control cell lines resulting in increased proliferation of growth. (PDF) [file pone.0259727.s004.pdf]

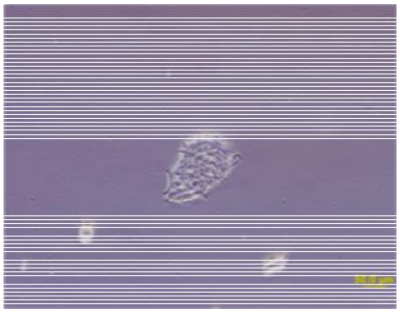

a: 293T-P2X4 cell line in presence of G418

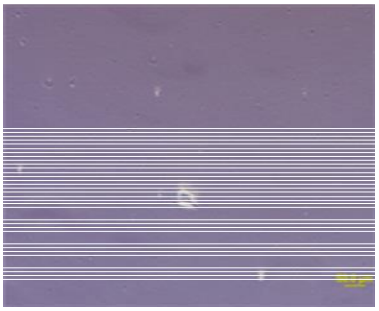

b: 293T cell line (control) in presence of G418

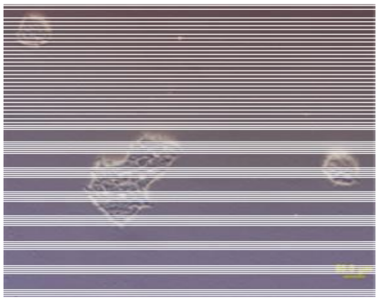

c: 293T- NV cell line in presence of G418

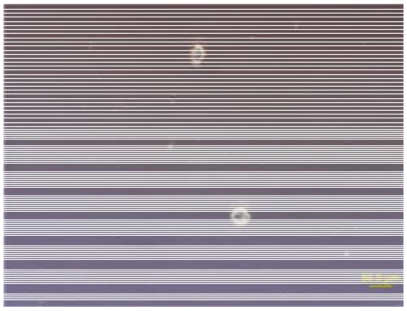

d: 293T cell line (control) in presence of G418

Figure (a,b,c,d): 293T/P2X4 cell line experimental and control in presence of G418 (a, b. 100X), 293T/NV cell line experimental and control in presence of G418(c, d. 100X). 293T/P2X4 cell line and 293T/NV cell line demonstrated better resistance to G418 in comparision to both control cell lines resulting in increased proliferation of growth.
