# Supplementary material for: P2X4 receptors mediate induction of antioxidants, fibrogenic cytokines and ECM transcripts; in presence of replicating HCV in in vitro setting: An insight into role of P2X4 in fibrosis
Source: PLoS One. 2022 May 20;17(5):e0259727. doi: 10.1371/journal.pone.0259727 (PMC9122194; doi:10.1371/journal.pone.0259727)
Supplement: S5 File — (PDF) [file pone.0259727.s005.pdf]

# 3. MATERIALS AND METHODS

## 3.1: Cell lines and culture conditions:

The cell lines used in this study, Huh-7 and Huh-7/E1E2. were kindly provided by Dr. Zafar Nawaz (Biochemistry and Molecular Biology Department, University of Miami, USA) and Dr. Muhammad Idrees (Incharge Molecular Virology Division, CEMB, university of The Punjab, Lahore, Pakistan) respectively. The Huh-7/E1E2 cell line was derived from the parental Huh-7 cell line (Human hepatocellular carcinoma or hepatoma cell line).The Huh-7/E1E2 cell line is a stable clone of Huh-7 cells transfected with and stably overexpresing HCV-Str. proteins E1E2 (derived from local HCV isolates of genotype 3a) linked to the antibiotic selection marker G418 which was under the control of human cytomegalovirus promoter. Huh-7 cell line was cultured in Dulbecco Modified Eagle's Medium (DMEM) supplemented with 100U/ml of penicillin and 100ug/ml of streptomycin and 10% heat inactivated fetal bovine serum (complete DMEM medium). Huh-7/Str cell line was maintained in complete medium containing 500µg/ml G418. The culture medium was renewed by a fresh medium every third day and cells were subcultured when they reached 70% confluency. All cells were maintained at 37°C in a humidified environment containing 5% $CO_2$ in a cell culture incubator.

## 3.2: RNA Extraction, quantification, and cDNA Synthesis:

Briefly, equal numbers of cells of both cell lines were plated in $25cm^2$ culturing flasks (6 different flasks, 3 for each) at the same time and kept at 37°C incubator in a humid air with 5% $CO_2$. On 3day, media was removed; cells were washed with sterilized 1X PBS (Phosphate Buffer Saline), trypsinized using 0.5% trypsin in EDTA. Total cellular RNA was extracted from the cells (Huh-7 and Huh-7/Str) using Gentra RNA Isolation Kit (Puregene, Minneapolis, MN 55441, USA), following the manufacturer's instructions. Each sample of isolated RNA was further treated with DNase (Fermentas) to remove any residual DNA present that could generate false -positive results. Quantity and quality of extracted RNA was assessed using NanoDrop® (Spectrophotometer) (ND-1000). Quality of extracted RNA was also checked on (1µg of RNA+0.8µl 6X DNA loading dye+ 1µl DEPC treated water) agarose gel electrophoresis using

1% agarose gel, stained with ethidium bromide and photographed. Best quality RNA samples were selected for cDNA synthesis. cDNA was generated using RevertAid$^{TM}$ H Minus First Strand cDNA synthesis kit (Fermentas, Cat no.K1632) according to the following protocol. All the tubes, pipette tips and containers used in the RNA extraction and cDNA synthesis were either manufacturer certified RNAase free or were made RNAase free by treating with 1% DEPC treated water and were autoclaved before use.

**Protocol of RT-PCR**

Following reaction mixture was prepared in a reaction tube while incubated in ice:

• Total extracted cellular RNA……………….. 1µg-5µg (1-5 µl)

• Oligo(dT)$_{18}$ Primer (0.5 µg/ µl)……………….1 µl

•DEPC treated water ......... make up volume 12µl

• The above reagents were mixed gently and spun down for 3-5sec. in a microcentrifuge.

• Incubated the mixture at 70°C in a thermocycler for 5min, chilled on ice and centrifuged briefly.

• Placed the reaction tube on ice and added the following components:

• 5X reaction Buffer…………………………………. 4µl

•RiboLock$^{TM}$ Ribonuclease Inhibitor (20u/ µl)……...1µl

•10Mm dNTP mix ………………………………...2µl

• Mixed gently and centrifuged briefly, incubated at 37°C for 5min.

• Added RevertAid$^{TM}$ H Minus M-MuLV……1µl

Final Volume ………. 20 µl

The cycling conditions for RT-PCR were as follows:

Incubated the mixture at 42 °C for 60 min and then stopped the reaction by heating at 70°C for 10 min Spun down and stored at - 20°C.

## 3.3: Designing of PCR primers for different markers:

Sense and anti-sense primers for the amplification of different markers for human antioxidant gene expression (CuZn/SOD, HO-1), proinflammatory cytokine (TNF-α), profibrotic cytokine (TGF-β1), vasoactive cytokine (Angiotensin II), adipokine (leptin) and GAPDH were designed using Primer3 software (http://bioinformatics.weizmann.ac.il/cgi-bin/primer/primer3.cgi) and were synthesized by the core facility of the centre (CEMB).

The sequences of gene specific primers were used in the study for PCR amplifications from cDNA are given in table 1.

| S.No | Primer Name | Primer Sequence: 5'-3' Sequence |
|---|---|---|
| 1 | Angiotensin II-F | CACGCTCTCTGGACTTCACA |
| 2 | Angiotensin II-R | GCTGTTGTCCACCCAGAACT |
| 3 | TGF- β1-F | TATCGACATGGAGCTGGTGA |
| 4 | TGF-β1-R | TGGGTTTCCACCATTAGCAC |
| 5 | HO-1-F | AGGTCATCCCCTACACACCA |
| 6 | HO-1-R | GTTGGGGAAGGTGAAGAAGG |
| 7 | Leptin-F | ACGTGCTGGCCTTCTCTAAG |
| 8 | Leptin-R | ACCTGGAAGCCAGAGTTCCT |
| 9 | Cu/Zn SOD-F | GGGGAAGCATTAAAGGACTG |
| 10 | Cu/Zn SOD-R | AATAGACACATCGGCCACAC |
| 11 | TNF -α-F | TCCTTCAGACACCCTCAACC |
| 12 | TNF -α-R | CAGGGATCAAAGCTGTAGGC |
| 13 | GAPDH-F | ACCACAGTCCATGCCATCAC |
| 14 | GAPDH-R | TCCACCACCCTGTTGCTGTA |

**Table 1: List of Primer sequences used in the current study**

## 3.4. Optimization of PCR Amplification:

Reaction conditions and PCR profile were firstly optimized for all primers for conventional PCR and finally for real time PCR using 1 µg concentration of cDNA. Set up for PCR reaction was as follows:

### 1: Optimization of Angiotensin II:

a. 2X fermentas master mixture …………………10µl
b. Forward Primer (5 pmol/ul) ……………………1µl
c. Reverse Primer (5 pmol/ul) ………….………….1µl
d. cDNA used (1µg/µl)…………………….…….. 1µl
e. Spermine ……………………...…………......1.25µl
f. Nuclease free water…………….…………...5.75µl

Total Reaction Volume = 20 µl

The tubes were placed on a PE-2700 (ABI) thermal cycler. The samples were preheated at 94°C for 45seconds and then run 35 cycles with the following parameters: at 94°C for 1 minute, 59°C for 45 seconds and 72°C for 1 minute. Final extension was done for 10 minutes at 72°C. PCR product was checked on agarose gel electrophoresis using 2% agarose. The gel was stained with ethidium bromide and photographed using gel documentation system. A 100 or 50-bp ladder (Fermentas) was run in each gel as DNA size marker. The amplified PCR product of expected size (209 bp) was obtained.

**2: Optimization of Leptin:**

a. 2X Fermentas master mixture . ……………. 10µl
b. Forward Primer (5 pmol/ul) …………………1µl
c. Reverse Primer (5 pmol/ul) …………..……….1µl
d. cDNA used (1µg/µl)…………………..…… 1µl
e. Spermine………… ………………………..1.25µl
f. Nuclease free water …………..………….. 5.75µl

Total Reaction Volume = 20 µl

The tubes were placed on a PE-2700 (ABI) thermal cycler. The samples were preheated at 94°C for 45seconds and then run 35 cycles with the following parameters: at 94°C for 1 minute, 57°C for 45 seconds and 72°C for 1 minute. Final extension was done for 10 minutes at 72°C. PCR product was checked on agarose gel electrophoresis using 2% agarose. The gel was stained with ethidium bromide and photographed using gel documentation system. A 100 or 50-bp ladder (Fermentas) was run in each gel as DNA size marker. The amplified PCR product of expected size (241bp) was obtained.

**3: Optimization of TGF-β1:**

a. 2X Fermentas master mixture ………………. 10µl
b. Forward Primer (5 pmol/ul) ……………………1µl
c. Reverse Primer (5 pmol/ul) ……..……………….1µl
d. cDNA used (1µg/µl)………………………… 1µl
e. Spermine…………………………………..1.25µl
f. Nuclease free water …………………….. 5.75µl

Total Volume = 20 µl

The tubes were placed on a PE-2700 (ABI) thermal cycler. The samples were preheated at 94°C for 45seconds and then run 35 cycles with the following parameters: at 94°C for 1

minute, 58°C for 45 seconds and 72°C for 1 minute. Final extension was done for 10 minutes at 72°C. PCR product was checked on agarose gel electrophoresis using 2% agarose. The gel was stained with ethidium bromide and photographed using gel documentation system. A 100 or 50-bp ladder (Fermentas) was run in each gel as DNA size marker. The amplified PCR product of expected size (246bp) was obtained.

## 4: Optimization of Cu/Zn-SOD:

a. 10X PCR Buffer $(NH4)_2SO_4$-$MgCl_2$……………………… 2µl
b. $MgCl_2$ (25mM)……………………………………………..2.4µl
c. dNTPs (500 µM)…..………………………………………1µl
d. *Taq*. DNA Polymerase(fermentas) (5U/µl)….……………..0.4µl(2U)
e. Forward Primer (5 pmol/ul)…………………………………1µl
f. Reverse Primer (5 pmol/ul)…………………………………..1µl
g. cDNA used (1µg/µl)……………………….…………………1µl
h. Nuclease free water ……………………... …………. 11.2µl

Total Reaction Volume = 20 µl

The tubes were placed on a PE-2700 (ABI) thermal cycler. The samples were preheated at 94°C for 45seconds and then run 35 cycles with the following parameters: at 94°C for 1 minute, 55°C for 45 seconds and 72°C for 1 minute. Final extension was done for 10 minutes at 72°C. PCR product was checked on agarose gel electrophoresis using 2% agarose. The gel was stained with ethidium bromide and photographed using gel documentation system. A 100 or 50-bp ladder (Fermentas) was run in each gel as DNA size marker. The amplified PCR product of expected size (203bp) was obtained.

## 5: Optimization of HO-1:

a. 2X Fermentas master mixture …………………………… 10µl
b. *Taq*. DNA Polymerase(fermentas) (5U/µl) ………….…….0.1µl(0.5U)
c. Forward Primer (2.5 pmol/ul) ……………………………0.5µl
d. Reverse Primer (2.5 pmol/ul) ……………………………..0.5µl
e. cDNA used (1µg/µl)………………………………………. 1µl
f. Spermine …………………………………. …………….1.25µl
g. Nuclease free water ……………………………..….. 6.65µl

Total Reaction Volume = 20 µl

The tubes were placed on a PE-2700 (ABI) thermal cycler. The samples were preheated at 94°C for 45seconds and then run 35 cycles with the following parameters: at 94°C for 1 minute, 58°C for 45 seconds and 72°C for 1 minute. Final extension was done for 10 minutes at 72°C. PCR product was checked on agarose gel electrophoresis using 2% agarose. The gel was stained with ethidium bromide and photographed using gel documentation system. A 100 or 50-bp ladder (Fermentas) was run in each gel as DNA size marker. The amplified PCR product of expected size (206bp) was obtained.

## 6: Optimization of TNF-α:

a. 2X Fermentas master mixture ………….……. 10µl
b. Forward Primer (5 pmol/ul) ……………………1µl
c. Reverse Primer (5 pmol/ul) ……….……………1µl
d. cDNA used (1 µg/µl)…………………….……… 1µl
e. Nuclease free water ……………………...….. 7µl

Total Reaction Volume = 20 µl

The tubes were placed on a PE-2700 (ABI) thermal cycler. The samples were preheated at 94°C for 45seconds and then run 35 cycles with the following parameters: at 94°C for 1

minute, 56°C for 45 seconds and 72°C for 1 minute. Final extension was done for 10 minutes at 72°C. PCR product was checked on agarose gel electrophoresis using 2% agarose. The gel was stained with ethidium bromide and photographed using gel documentation system. A 100 or 50-bp ladder (Fermentas) was run in each gel as DNA size marker. The amplified PCR product of expected size (208bp) was obtained.

## 7: Optimization of GAPDH:

a. 2X Fermentas master mixture ……..……… 10µl
b. Forward Primer (5 pmol) ………..…………0.5µl
c. Reverse Primer (5 pmol) ……………….….0.5µl
d. cDNA used (1µg/µl)………….………..….… 1µl
e. Nuclease free water ……………………….. 8µl

Total Reaction Volume = 20 µl

The tubes were placed on a PE-2700 (ABI) thermal cycler. The samples were preheated at 95°C for 5mintutes and then run 35 cycles with the following parameters: at 94°C for 30 seconds, 58°C for 40 seconds and 72°C for 40 seconds. Final extension was done for 7 minutes at 72°C. PCR product was checked on agarose gel electrophoresis using 2% agarose. The gel was stained with ethidium bromide and photographed using gel documentation system. A 100 or 50-bp ladder (Fermentas) was run in each gel as DNA size marker. The amplified PCR product of expected size (453bp) was obtained.

## 3.5. Quantitative Real Time PCR.

Reaction conditions and PCR optimization was done for all primers with real time PCR.

### 3.5.1. Optimization of Angiotensin II, Cu/Zn-SOD, TNF-α, TGF-β and Leptin:

a. 2X Maxima™ SYBR Green qPCR Master Mix …….12.5µl
b. Forward Primer (6.4 pmol/ul) ………….…………1.6µl
c. Reverse Primer (6.4 pmol/ul) ………………….……...1.6µl
d. cDNA used (1µg/µl)…………………………………. 1µl
e. Nuclease free water ………………………..……. 8.3µl

Total Reaction Volume = 25µl

### 3.5.2. Optimization of HO-1 for qRT-PCR:

a. 2X Maxima™ SYBR Green qPCR Master Mix ………………. 12.5µl
b. Forward Primer (3.2pmol/ul) ……………………………………0.8µl
c. Reverse Primer (3.2pmol/ul) …………………………………...0.8µl
d. cDNA used (1µg/µl)……………………………………..………. 1µl
e. Nuclease free water ……………………………………………. 9.9µl

Total Reaction Volume = 25 µl

### 3.5.3. Optimization of GAPDH for qRT-PCR:

a. 2X Maxima™ SYBR Green qPCR Master Mix …………. 12.5µl
b. Forward Primer (8pmol) ……………………………………0.8µl
c. Reverse Primer (8pmol) …………………….……….......0.8µl
d. cDNA used (1µg/µl)……………………...………………. 1µl
e. Nuclease free water …………………………….…………. 9.9µl

Total Reaction Volume = 25 µl

Real time PCR reactions were run on Cepheid smart cycler II (France) using PCR profiles that were used to optimized PCR products at conventional PCR (PE-2700 (ABI) (thermal cycler). However, final extension was excluded in PCR profile as there is no need of final extension in Real time PCR system.

# PART-I

## 3.6. Cu/Zn-SOD, HO-1, TNF-α, TGF-β, Leptin and Angiotensin II gene Expression in Huh-7 and Huh-7 Str. Cell lines by Quantitative Real Time PCR:

Real time qRT-PCR was performed to examine the mRNA expression of various fibrosis inducing factors as Cu/Zn-SOD, HO-1, TNF-α, TGF-β, Leptin and Angiotensin II in Huh-7/E1E2 cell line compared to wild type/parental Huh-7 cells as control. Equal numbers of cells of both cell lines were plated in 25cm$^2$ culturing flasks (6 different flasks, 3 for each) at the same time and kept at 37°C incubator in humid air with 5% $CO_2$. On 3day, media was removed; cells were washed with sterile 1X PBS (Phosphate Buffer Saline), trypsinized using 0.5% trypsin in EDTA. Cells were centrifuged at 5,000 RCF for 3 mints and total cellular RNA was extracted from the cells (Huh-7 and Huh-7/Str) using Gentra RNA Isolation Kit (Puregene, Minneapolis, MN 55441, USA), following the manufacturer's instructions. Quantity and quality of extracted RNA was assessed NanoDrop® (Spectrophotometer) (ND-1000). Quality of extracted RNA also checked on running it in agarose gel electrophoresis unit using 1% agarose gel, stained with ethidium bromide and photographed. Highly quality RNA samples were selected for cDNA synthesis. cDNA was generated using RevertAid™ H Minus First Strand cDNA synthesis kit (Fermentas, Cat no.K1632) according to following protocol described previously (in section 3.2). PCR was performed to determine the gene expression level. PCR products were checked on agarose gel electrophoresis using 2% agarose stained with ethidium bromide to specify amplification. Then Real Time PCR reactions were run on Cepheid smart cycler II (France) (Reaction conditions and PCR profile have been described previously in sec.3.5). The relative levels of Cu/Zn-SOD, HO-1, and TNF-α, TGF-β, Leptin and Angiotensin II genes were determined using GAPDH mRNA as control. The calculation was based on the Δ-Ct (Threshold cycle number difference between control, Huh-7, and Huh-7/E1E2cells), and the ratios were normalized to the ratios of GAPDH of the corresponding samples.

## 3.7. Identification and Expression of Different Isoforms of P2X receptors In Huh-7 and Huh-7/E1E2 Cell Lines

### 3.7.1. Culture, RNA isolation and cDNA synthesis of Huh-7 and Huh-7 E1E2 Cell lines:

Huh-7 cell line was cultured in DMEM (complete medium) supplemented with 10% FBS. Huh7/E1E2 cell line was cultured in DMEM (complete medium) supplemented with 10% FBS and 500µg/ml G418 (described in sec.3.1). RNA isolation and cDNA synthesis from both cell lines were done by same procedures as described in sec.3.2.

### 3.7.2. PCR Amplification:

P2X receptors display a diverse tissue distribution (Young *et al.,* 2008). Currently seven isoforms of P2X receptors have been cloned and characterized (Burnstock, 2007). We identified six different isoforms of P2X receptors (P2X2, P2X3, P2X4, P2X5 and P2X6) in Huh-7 cell line (Human hepatocellular carcinoma or hepatoma cell line).

RT-PCR (PCR amplification) experiments were repeated using at least three separate preparations of synthesized cDNA, from at least three separate experiments to confirm the consistency of results obtained. Two different concentrations of cDNA (1µg & 5µg) were used for optimization of PCR amplification, so that if any isoform has a faint expression in Huh-7 cell line, then that could also be detected at 5µg/µl cDNA concentration.

Previously published primer sequences were used for isoforms, P2X2, P2X3, P2X5 (in human cervical epithelial cells, Gorodeski et al., 2002, in human intestinal epithelial carcinoma cells, Coutinho-Silva et al., 2005). For P2X6 receptor, independent sense and antisense primers were designed from known cloned receptor sequence, available in GenBank using primer 3 software, and were synthesized by the core synthesis facility of the centre (CEMB). We did not identify P2X1 and P2X7 in Huh-7 cell line. The specific primer sequences used in present study are given below:

| S.No | Primer Name | Primer Sequence: 5'-3' Sequence |
|---|---|---|
| 1 | P2X2 F | CAGGTTTGCCAAATACTACAAGATCA |
| 2 | P2X2 R | AACTTCCCGGCCTGTCCAT |
| 3 | P2X3 F | TCTTCACCTATGAGACCACCAAGTC |
| 4 | P2X3 R | GATCAGAAGCTGAACTACTCGGTTGATG |
| 5 | P2X4 F | CTC TGCTTGCCCAGGTACTC |
| 6 | P2X4 R | CCAGCTCACTAG CAAGACCC |
| 7 | P2X5 F | CGCTGGGGAAGCGGTTA |
| 8 | P2X5 R | GCACCAGGCAAAGATCTCACA |
| 9 | P2X6 F | GAACCACAATTCAGCCCCTA |
| 10 | P2X6 R | CAGGTCACAATCCCAGTGAA |

**Table 2: List of Primer sequences used for amplification of P2X receptors isoforms**

**3.7.2.1. PCR Mix Preparation:**

**For amplification of P2X2, P2X3, P2X4 and P2X5:**

a. 2X fermentas master mixture …………… ……..10µl
b. Forward Primer (5pmol/ul) ………………………..1µl
c. Reverse Primer (5pmol/ul) ……….....………………1µl
d. cDNA used (1µg/µl or 5 µg/µl)………...…………… 1µl
e. Nuclease free water …………………...………….7µl

Total Reaction Volume = 20 µl

PCR conditions for amplification of P2X2, P2X3 and P2X5 were 90°C (10 minutes), 40 cycles [95°C (45 seconds), 60°C (45 seconds) and 72°C (45 seconds)], and 72°C (10 minutes), PCR conditions for P2X4 were 94°C for 45seconds 35 cycles [94°C (1minute), 60°C (45

seconds) and 72°C (1 minute)], and 72°C (10 minutes), for P2X7 90°C (10 minutes), 40 cycles [95°C (45 seconds), 59°C (45 seconds) and 72°C (45 seconds)], and 72°C (10 minutes).

**For amplification of P2X6:**

a. 10X PCR Buffer $(NH4)_2SO_4$-$MgCl_2$…………………………. 2µl
b. $MgCl_2$ (25mM) ……………………….. …………………2.4µl
c. dNTPs (500 µM) ………………………………..…………1µl
d. *Taq*. DNA Polymerase(fermentas) (5U/µl) ……….………. 0.4µl(2U)
e. Forward Primer (7.5pmol/ul) ……………………………..1.5µl
f. Reverse Primer (7.5pmol/ul) ………………..…………..1.5µl
g. cDNA used (1µg/µl or 5µg/µl)………………..……………1µl
h. Nuclease free water ……………….... ………………. 10.2µl

Total Reaction Volume = 20 µl

PCR conditions for amplification of P2X6 were 94°C (45 seconds), 35 cycles [94°C (1minute), 59°C (45 seconds) and 72°C (1 minute)], and 72°C (10 minutes).

**3.7.3. Purification of PCR products:**

Amplified PCR products were separated by gel electrophoresis (2% agarose) and visualized with ethidium bromide staining. A 100 or 50-bp ladder (Fermentas) was run in each gel as DNA size marker. The amplified products of P2X2, P2X3, P2X4, P2X5 and P2X6 were carefully excised from the gel, using a sterilized surgical blade and transferred into a sterile eppendorf. These were eluted from agarose gel using gel extraction kits (Fermentas or QIAEX II gel extraction kit (Qiagen, Valencia, CA). Amplified products of P2X2, P2X3 and P2X5 were eluted using QIAEX II Gel Extraction Kit (Specific for amplified products less than 100 bp) according the kit protocol. While amplified products of P2X4 and P2X6 were eluted using Fermentas gel extraction kit (Specific for amplified products above than 100 bp) by following the manufacturer's instructions.

Eluted DNA was run on 2% agarose gel along with 100 or 50 bp ladder (Fermentas) as a marker for confirmation and estimation of PCR products. Further the amount of purified DNA was also quantified using NanoDrop® (Spectrophotometer) (ND-1000). The identity of all amplified products was confirmed by sequencing using an automated sequencer.

### 3.7.4. Sequencing PCR:

The purified DNA of P2X2, P2X3, P2X4, P2X5 and P2X6 were used as templates for sequencing PCR in the Big-Dye Terminator Cycle Sequencing Ready Reaction Kit (Applied Biosystems). Samples were analyzed on an automated sequencer (ABI PRISM 3100 genetic analyzer; Applied Biosystems). Products were sequenced from both strands to confirm their identity with reference sequences.

**Preparing Sequencing Reactions**

- For P2X2 added the following reagents in a reaction tube:
  a. DNA template (5ng/µl) =…………………1µl
  b. Big Dye =……………………………… 0.5µl
  c. 5X Sequencing Buffer =……………..1.75µl
  d. R/F Primer (10pmol/µl) =………………..1µl
  e. Nuclease free water = ………………..5.75 µl

Total Reaction Volume = 10 µl

PCR conditions for both forward and reverse amplification were 96°C (1 minute), 25cycles [96°C (30 seconds), 57°C (15 seconds) and 60°C (4 minutes)], and 60°C (4 minutes).

- For P2X3 added the following reagents in a reaction tube:
  a. DNA template (3ng) =…………………… 0.3µl
  b. Big Dye =………………………………..0.3µl
  c. 5X Sequencing Buffer =…………………1.90µl
  d. R/F Primer (10pmol/µl) =……………………1µl
  e. Nuclease free water =…………………….. 6.6 µl

Total Reaction Volume = 10 µl

PCR conditions for both forward and reverse amplification were 96°C (1 minute), 25cycles [96°C (30 seconds), 57°C (15 seconds) and 60°C (4 minutes)], and 60°C (4 minutes). For reverse amplification annealing temperature was 55°C (15 seconds).

● For P2X4 and P2X5 added the following reagents in a reaction tube:

a. DNA template (10ng/ µl) =…………………5µl
b. Big Dye =………………………………...1µl
c. 5X Sequencing Buffer =……………………2µl
d. R/F Primer (10pmol/µl) =………….……..1µl
e. Nuclease free water =………………………1µl

Total Reaction Volume = 10 µl

PCR conditions for P2X4 were 94°C for 45seconds 30 cycles [94°C (1minute), 60°C (45 seconds) and 72°C (1 minute)], and 72°C (10 minutes). PCR conditions for P2X5 were 90°C (10 minutes), 30 cycles [95°C (45 seconds), 60°C (45 seconds) and 72°C (45 seconds)], and 72°C (10 minutes).

● For P2X6 added the following reagents in a reaction tube:

a. DNA template (10ng) =……………………3µl
b. Big Dye =……………………………...0.5µl
c. 5X Sequencing Buffer =…………………...2µl
d. R/F Primer (15 pmol/µl) =………………1.5µl
e. Nuclease free water =…………………….. 3µl

Total Reaction Volume = 10µl

PCR conditions for P2X6 were 94°C for 45seconds 25 cycles [94°C (1minute), 55°C (45 seconds) and 72°C (1 minute)], and 72°C (10 minutes).

### 3.7.5. Purifying Extension Products Using Ethanol Precipitation Method

The extension products were purified using ethanol precipitation method as described below:

a. Spun down the contents of the reaction tubes.
b. Added 16 µl of deionized water and 64 µl of non-denatured 95% ethanol. Every time the final concentration was 60 $\pm$ 3%.
c. Closed the tubes and vortexed briefly.
d. Left the tubes at room temperature for 15 minutes to precipitate the extension products.
e. Spun down the tubes for 20 minutes at 12000 x g.
f. Aspirated the supernatant carefully with a separate pipette tip for each sample and discarded it.
g. Added 250 µl of 70% ethanol to the tubes and mixed well.
h. Spin down for 10 minutes at the same orientation.
i. Aspirated the supernatant carefully.
j. Dried the samples in dark.

### 3.7.6. Sample Electrophoresis

a. Re-hydrated the pellet in 15 µl formamide and mixed well by up/down pipetting. The samples were shifted in 96-wells plate. Heat denatured at 95 ºC for 5 minutes in thermal cycler and immediately put on ice for 10 minutes.
b. The sequenced samples with BigDye terminators were electrophoresed on ABI PRISM 3100 sequencer genetic analyzer that is equipped with required modules and dye set/primer files.
c. Sequences were analyzed manually by using Chromas software version (v 1.45).

Homology studies of the nucleotide sequences of amplified and sequenced P2X isoforms with known nucleotide sequence present in gene data bank was done through standard nucleotide–nucleotide Blast (Basic Local Alignment Search Tool) software available at NCBI website <http://www.ncbi.nlm.nih.gov/BLAST>

Nucleotide sequences were put in FASTA format and with the help of the BLAST2 SEQUENCE SOFTWARE; pair wise comparisons were done for percent nucleotide homology. Sequences showed 96% (for P2X2) to 100% (for P2X3, P2X4, P2X5, P2X6) homology with reported sequences.

### 3.7.7. Quantitative Real Time PCR:

**●Optimization of P2X2, P2X3, P2X4, P2X5, for qRT-PCR:**

a. 2X Maxima™ SYBR Green qPCR Master Mix ………………12.5µl
b. Forward Primer (6.4pmol/ul)……………………………………1.6µl
c. Reverse Primer (6.4pmol/ul) ……………………………….....1.6µl
d. cDNA used (1µg/µl)…………………………………….…. 1µl
e. Nuclease free water ……………………………………… 8.3µl

Total Reaction Volume = 25µl

**●Optimization of P2X6 for qRT-PCR:**

a. 2X Maxima™ SYBR Green qPCR Master Mix ……………. 12.5µl
b. Forward Primer (9.6pmol/ul)……………………………………2.4µl
c. Reverse Primer (9.6pmol/ul) ………………………………...2.4µl
d. cDNA used (1µg/µl)…………………………………….…. 1µl
e. Nuclease free water ……………………………………… 6.7µl

Total Reaction Volume = 25 µl

Real time PCR reactions were run on Cephid Real time system at same PCR profiles which were used to optimized PCR products at conventional PCR (PE-2700 (ABI) thermal cycler). However, final extension was excluded in PCR profile as there is no need of final extension in Real time PCR system.

**3.7.8. P2X receptors gene Expression in Huh-7 and Huh-7/E1E2. Cell lines by Quantitative Real Time PCR:**

Real time qRT-PCR was performed to examine the mRNA expression of identified isoforms of P2X receptors in Huh-7/E1E2 cell line compared to wild type/parental Huh-7 cells as control. Equal numbers of cells of both cell lines were plated in 25cm$^2$ culturing flasks (6culturing flasks , 1 for each cell line in 3 separate studies) at the same time and kept at 37°C incubator in humid air with 5% $CO_2$. On 3day, media was removed; cells were washed with sterile 1X PBS (Phosphate Buffer Saline), trypsinized using 0.5% trypsin in EDTA. The RNA extractions and cDNA symthesis were done as described previously.

Then real time PCR reactions were run on Cepheid smart cycler II (France) (Reaction conditions and PCR profile have been described previously in sec.3.6.7). The relative levels of P2X2, P2X3, P2X4, P2X5, P2X6 genes were determined using GAPDH mRNA for normalization. The calculation was based on the Δ-Ct (Threshold cycle number difference between control, Huh-7, and Huh-7/E1E2 cells), and the ratios were normalized to the ratios of GAPDH of the corresponding samples. Some isoforms were unresponsive to HCV STR. Proteins E1E2, while some were responsive (See Results). The most responsive isoform was P2X4. Therefore, P2X4 was cloned in mammalian expression vector pcDNA3.1+. 293T cells were transfected with pcDNA3.1+/ P2X4 and pcDNA3.1+ alone. Two different cell lines stably expressing P2X4 and pcDNA3.1+ were established (293T/P2X4 and 293T/ pcDNA3.1+) and successfully infected with human HCV serum, to further examine the role of P2X4 signaling in the induction of fibrosis.

# PART-II

## CLONING OF P2X4 AND DEVELOPMENT OF CELL CULTURE BASED SYSTEM USING VECTOR CARRYING P2X4 AND HUMAN HCV SERUM

### 3.8. Sub cloning of P2X4 in a mammalian expression vector pcDNA3.1+(Construction of Expression plasmid)

Full length P2X4 (1.7kb) cloned in plasmid pCR 3.1(pCR3.1/P2X4) was kindly provided by Dr. Ishtiaq Qadri (Director, NCVI, National University of Science and Technology, Pakistan). P2X4 was excised from vector pCR 3.1 and cloned in vector pcDNA3.1+ to obtain expression plasmid, P2X4/ pcDNA3.1+.

The steps involved in cloning are given below:

**a) Competent cells preparation:**

Many *E. coli* strains such as JM109, DH5α and BL21 (DE3) PlySE, were used for various plasmid and ligation mixture transformations. Before this, competent cells of the *E. coli* strains (DH5α) were prepared for high efficiency transformation. A single colony from overnight grown LB plate was inoculated into 3ml of LB broth medium in a 15ml culture tube and incubated with vigorous shaking at 37ºC overnight. One ml of overnight grown culture was diluted in 500 ml of SOB medium in a 2 liter flask and incubated for 2-3 hours with vigorous shaking at 37ºC until O.D. was 0.8 at 595nm. Cells were harvested by centrifugation at room temperature and pellet was washed twice by resuspending in ice cold 10% sterile glycerol solution. The cell pellet was then resuspended in 1ml of 10% sterile glycerol. Cells were aliquoted in 50µl aliquots and immediately stored at –70ºC for future use.

**b) Restriction digestion of pCR/P2X4 and pcDNA3.1+:**

The pure empty mammalian expression vector pcDNA3.1/Zeo$^R$(+) (Invitrogen,, Life Technologies) and vector carrying insert pCR3.1/P2X4 were linearized by digestion with NOT1 and HindIII restriction enzymes to provide sticky ends for ligation of P2X4 into pcDNA3.1+.

**1: Digestion Mix: pcDNA3.1+ (Pure empty vector without any insert)**

The following materials were added in reaction tube and incubated at 37$^{o}$C for 1.5 hrs. The reaction mixture was as follows:

Plasmid DNA (pcDNA3.1+)…………………..30 µl
10x Tango yellow buffer………………………...3 µl
NOT1 (10U/µl)…………………………………….3µl
HindIII(10U/µl)……………………………………3µl
Double distilled water………………………….…21µl
Final Reaction Volume…………60 µl

The digested vector was run on 1% agarose gel in 1X TAE buffer, at constant 80V for 90 min and analyzed under UV-light. The gel slices containing digested product (pcDNA3.1+, 5kb) was cut with sterilized blade and were eluted according to the protocol of Fermentas DNA Extraction Kit (Cat # K0513) to elute the DNA. 5µl of purified vector and insert were run on 1% agarose gel with λHindIII digested marker to check concentration for further ligation.

**2: Digestion Mix: pCR/P2X4 (Vector carrying insert)**

The following materials were added in reaction tube and incubated at 37$^{o}$C for 1.5 hrs. The reaction mixture was as follows:

Plasmid DNA (pCR/P2X4)……………….. 40µl
10X Tango yellow buffer…………….…….. 4µl
NOT1 (10U/µl) ………………………………4µl
HindIII(10U/µl)……………………………… 4µl
Double distilled water……………………….28µl
Final Reaction Volume…………80µl

The digested vector was run on 1% agarose gel, 80V for 90 min and analyzed under UV-light. The gel slices containing digested product (P2X4, 1.7kb) was cut with sterilized blade and were purified according to the protocol of Fermentas DNA Extraction Kit (Cat # K0513). 5µl

of purified vector and insert were run on 1% agarose gel with λHindIII digested marker to check concentration for further ligation.

**c) Ligation of Insert (P2X4) into Vector pcDNA3.1+:**

Digested pcDNA3.1+ was ligated with insert P2X4 in different ratios 1:1, 1:3 and 3:1 and incubated at $14^0$C overnight. Reaction mixtures were as follows:

1. **Reaction MIX. (1:1)**

Plasmid DNA (pcDNA3.1+) …………..5µl
Insert DNA(P2X4)……………………...2µl
T4 DNA ligase…………………….……1µl
10X ligation buffer……………………..1µl
Double distilled water…………………..6µl
Final Reaction Volume……………15µl

2. **Reaction MIX. (1:3)**

Plasmid DNA (pcDNA3.1+) …………..5µl
Insert DNA(P2X4) ……………..………5µl
T4 DNA ligase…………………….……1µl
10X ligation buffer……………………. 1µl
Double distilled water …………………..3µl
Final Reaction Volume…………15µl

3. **Reaction MIX. (3:1)**

Plasmid DNA (pcDNA3.1+) ………….10µl
Insert DNA(P2X4) …………………...….3µl
T4 DNA ligase……………………..……2µl
10X ligation buffer…………………….. 2µl
Double distilled water………………….. 3µl
Final Reaction Volume…………20µl

**d) Heat shock transformation**

Ligation product of pcDNA 3.1+ and P2X4 was transformed into DH5α competent cells through heat shock method. For this purpose 10 µl of ligation product was added into 50ul of competent cells and mixed well while keeping it on ice. Cells were placed on ice for 30 min. The water bath was set at 42$^{o}$C and heat shock was given to the cells for 90 sec. Cold it again on ice for 3 min. and 400 µl of SOC medium added to each tube. Grew for 45 min at 37$^{o}$C with shaking and 200 µl of the growth was spread on L.B/AMP plates and incubated at 37$^{o}$C overnight (16 hours). Transformed colonies appeared after 12 to 16 hrs.

**e) Plasmid DNA isolation (Mini prep):**

Plasmid DNA psP2X4 was prepared by inoculating single bacterial colony in 3ml LB broth containing the ampicillin 100µg/ml from the overnight grown plate. The samples were incubated at 37ºC at 250 rpm for 12-16 hours. The plasmid DNA was isolated using GeneJET $^{TM}$ Plasmid miniprep kit (Fermentas) following the porotocol provided with kit.

**f) Quantification of plasmid DNA:**

To quantify the isolated plasmid DNA, its concentration was measured with nanodrop and gel electrophoresis. 5µl of isolated and purified DNA was run on 1% agarose gel with λHindIII digested marker to estimate the concentration of DNA.

**g). Confirmation of the cloned psP2X4:**

**1. Analysis by Restriction digestion:**

The double digestion of constructed vector psP2X4 was done with *NOT1* and *Hind III* confirmed the size of plasmid made.

The following materials were added in reaction tube and incubated at 37$^{o}$C for 1and 1/2hrs. The reaction mixture was as follows:

Plasmid DNA (psP2X4) …………..10µl

10X Tango yellow buffer……..……1µl

NOT1 (10U/µl)……………………...1µl

HindIII(10U/µl) ………………...…1µl

Double distilled water ……………...7 µl

Final ReactionVolume……….. 20 µl

The digested vector along with λHindIII digested marker was run on 1% agarose gel in 1X TAE buffer, at 80V for 90 min and analyzed under UV-light. Two bands (5.0kb and 1.7 kb) were obtained, showed the successful construct of expression vector of appropriate size. Furthermore, the confirmation of construct psP2X4 was done through sequencing PCR.

**2. Analysis by Sequencing PCR:**

All plasmid constructs isolated from a single colony, were sequenced for confirmation. Sequence analysis of the plasmid DNA was performed according to the manufacturer's instructions (Big Dye Deoxy Terminators; Applied Biosystems, Weiterstadt, Germany). Sequencing of both positive and negative strands on automated sequencer (Applied Biosystems 3100 DNA Analyzer) was performed.

The reaction mixture consisted of:

Big-Dye...……………………………0.6µl

5x sequencing buffer………………....2µl

T7 primer (10 pM)…………………1.0µl

Nuclease free wate…………………4.4µl

Template DNA (psP2X4)…………….2µl

Total Volume = 10µl

The cycling conditions for sequencing PCR were as 95°C (2 minutes), 30 cycles [95°C (50 seconds), 48°C (1 minute) and 60°C (4 minutes)], and 68°C (10 minutes).

After the sequencing PCR the PCR product was precipitated with ethanol as described previously in sec. 3.6.5. and 3.6.6. The deduced nucleotide sequence of the plasmid psP2X4 was

aligned with the P2X4 sequence (kindly provided by Ishtiaq Qadri Director, NCVI, National University of Science and Technology, Pakistan) using of Basic Local Alignment Search Tool (BLAST). It showed 100% homology with provided sequence of P2X4. After confirmation of successful construction of plasmid, high grade and pure plasmids psP2X4 and pcDNA3.1+ were isolated using Fermentas GeneJET ™ Plasmid miniprep kit and stored at -20°C for future use.

## 3.9. Establishment of stable cell lines:

Two different cell lines 293T/P2X4 (stably expressing P2X4 protein, Experimental), and 293T/pcDNA3.1+ or 293T/NV (vector alone without any insert/ null vector) were established to further study the role of P2X4 (which showed most response to the HCV structural proteins E1E2) in the presence of HCV all structural and non structural proteins by infecting them with human HCV serum (Buck, 2008). The steps involved are given below:

**A). Linearization of Plasmids:**

Plasmids, psP2X4 and pcDNA3.1+ (described previously in section 3.6) were linearized before transfection to facilitate their stable integration in 293T cells genome. Restriction enzyme *Bgl II* (Fermentas) was employed to linearized pcDNA3.1+/P2X4 and pcDNA3.1+. Before using *Bgl II*, it had been confirmed using soft ware NEBcutter V2.0 that this enzyme was quite safe for important sequences required for protein synthesis of inserted gene. *Bgl II* cut the pcDNA3.1+ only at single site (nucleotide number 13, far away from CMV promoter, see plasmid map; sec 3.6). *Bgl II* did not cut at any nucleotide site of inserted gene P2X4 (using soft ware NEBcutter V2.0). Plasmids were also quantified using NanoDrop® (Spectrophotometer) (ND-1000) to facilitate the optimization of linearization reaction. Following materials were added in two separate reaction tubes.

**Reaction MIX:**

**●Digestion of psP2X4, 6.7kb (pcDNA3.1+ P2X4, 5.0 kb+1.7kb):**

Pure Plasmid DNA psP2X4 (219.80ng/µl or 12 µg) …………..54 µl
Bgl II (10U/ µl) ………………………………………….……. 5 µl
10X Buffer O (Fermentas)………………...…………………. 7µl
Nuclease free water……………………...…………………4 µl

Total Volume = 70 µl

**●Digestion of pcDNA3.1+ (5.0 kb):**

Pure Plasmid DNA pcDNA 3.1+ (304ng/µl or 8 µg) …………..25 µl
Bgl II (10U/ µl) ………………………………………………… 3.2µl
10X Buffer O (Fermentas)……………………………………..5 µl
Nuclease free water…………………………………………….16.8 µl

Total Volume = 50 µl

Reaction tubes were incubated at 37°C for 2 hours, after that 3 µl of CIAP (Fermentas) was added in each reaction tube and again incubated at 37°C for 30 minutes.

**●Purification of Digested Plasmids:**

Digested plasmids were run on 1% agarose gel, 80V for 90 min and analyzed under UV-light. The gel slices containing linearized/ digested products (psP2X4 and pcDNA 3.1+) were cut with sterilized blade. Plasmids were purified according to the protocol of Fermentas DNA Extraction Kit (Cat # K0513). 5µl of purified plasmids were run on 1% agarose gel with λHindIII digested marker to check concentration for transfection. Concentration was also checked on nanodrop.

**B). Cell Line and Culture Condition:**

The cell line used in this part of study, HEK 293T (HEK, human embryonic kidney cell line) was kindly provided by Dr. Ishtiaq Qadri (Director, NCVI, National University of Science and Technology, Pakistan). 293T cell line was cultured in Dulbecco Modified Eagle's Medium (DMEM) supplemented with 100U/ml of penicillin and 100ug/ml of streptomycin and 10% heat inactivated fetal bovine serum (complete DMEM medium). After transfection with pcDNA3.1+ with a zeocin resistance cassette (Invitrogen) that contained full length P2X4 or vector control, 293Tcell lines were maintained in complete medium supplemented with 1mg/ml G418 for more than one month for the selection of stable clones. 293T stable cell lines were grown in complete DMEM supplemented with 500ug/ml G418. Cells were maintained at 37°C in a humidified environment containing 5% $CO_2$ in a cell culture incubator. The cells were subcultured from 1 culturing flask to 3 culturing flasks when confluence reached 70-80%, which was generally every 3 days.

**C). DNA Transfections:**

293T cells were prepared for transfection by plating onto 4 petri-dishes (60mm) at the time of subculture, 2days before transfection in DMEM containing appropriate suppliments. They were transfected with plasmid pcDNAP2X4 (experimental) and pcDNA3.1 vector alone (control). After 2days cells became 70-80% confluent and transfections were carried out using Lipofectamine™ 2000 (Invitrogen, Cat No.11668-019) following the manufacturer's instructions. Two different concentrations of purified, linearized plasmid DNA (2µg DNA+6µl Lipofectamine and 4µgDNA+10µl Lipofectamine) were used for optimization of transfection reaction. At 48 hrs posttransfection, cells were subcultured into 25cm$^2$ culturing flasks and 60mm petri-dishes and were grown in DMEM (complete culturing medium, Sec. 3.1) supplemented with selective agent G418 (1mg/ml).

**D). Selection of Stable Cell Lines:**

After successful transfections, cell lines 293T/P2X4 and 293T/Null vector (NV) were continuously grown in complete DMEM supplemented with G418 (1mg/ml). Cells were

subcultured on every day 4-5 depending upon confluency of cells. At the same time 293T cells without any transfection were also grown in the presence of selective reagent G418 (1mg/ml) as a control for optimization of selection of resistant stable clones. The cells in this flask (control) were also subcultured (depending upon confluency), old media was replaced with fresh one supplemented with G418 (1mg/ml). 293T cells in this control culture (without any transfection but continuously receiving selective agent G418 at the same concentration of transfected cell lines) were died after 20-25days. Transfected cell lines were grown in G418 (1mg/ml) continuously for more than 1 month (35d). After that, cells were splitted, approximately 25% grown for 2-3 day in the presence of G418 (1mg/ml). Single colonies were isolated using sterile filtered tips employed on sterile pippett, under microscope and sterile conditions. Single clones were transferred to 24- well culturing plates, grown in the presence of G418 (500μg/ml). After 15 days single clones were trypsinized and shifted into 6- well plates. Upon 70% confluence, single clones with stable expression were splitted into 25cm$^2$ culturing flasks. Expression of P2X4 was verified by reverse transcriptase PCR (mRNA) and western blotting (protein) and highest expressing clones were selected for further experiments.

**E). Verification of Stable Cell Lines:**

**A. Reverse Transcription –Polymerase Chain Reaction (RT-PCR) Analysis:**

RNA isolation and cDNA synthesis were performed following protocols previously described in Sec. 3.1. Previously reported primers were used for detection of transfected P2X4 mRNA (Doctor et al., 2004) while for detection of vector primers were designed at sequence T7 promoter and BGH reverse priming sit using Primer3 software (http://bioinformatics.weizmann.ac.il/cgi-bin/primer/primer3.cgi). Following primers were used for PCR amplifications from cDNA.

| S.No | Primer Name | Primer Sequence: 5'-3' Sequence |
|---|---|---|
| 1 | PX4T- F | CGTGGCGGACTATGTGATT |
| 2 | PX4T-R | GTGATGTTGGGGAGGATGTTC |
| 3 | T7 | GTAATACGACTCACTATAGGG |
| 4 | BGH | TAGAAGGCACAGTCGAGG |

**Table 3: List of Primer sequences used in the present study**

### 1: PCR Amplification of Transfected P2X4:

a. 2X fermentas master mixture ……………. 10µl
b. Forward Primer (5pmol/ul) ………….……1µl
c. Reverse Primer (5pmol/ul) ………………….1µl
d. cDNA used (1µg/µl)……………..…..…… 1µl
e. Nuclease free water ……………………..….. 7µl

Total Reaction Volume = 20µl

PCR conditions were 94°C (2 minutes), 35 cycles [94°C (30 seconds), 58°C (30 seconds) and 72°C (1 minute)], and 72°C (10 minutes). Amplification product was run on 2% agarose gel and visualized with ethidium bromide staining. A 100 or 50-bp ladder (Fermentas) was run as DNA size marker. Amplified product of expected size was obtained. The amplified product was carefully excised from the gel, using a sterilized surgical blade and transferred into a sterile eppendorf and eluted from agarose gel using gel extraction kits (Fermentas; Cat No.K0513) by following the manufacturer's instructions.

Eluted DNA was run on 2% agarose gel along with 100 or 50 bp ladder (Fermentas) as a marker for confirmation and estimation of PCR products. Further the amount of purified DNA was also quantified NanoDrop® (Spectrophotometer) (ND-1000).The identity of amplified product was confirmed by sequencing using an automated sequencer.

- **Sequencing PCR:**

The purified DNA of P2X4 (amplified from mRNA of stable cell line) was used as template for sequencing PCR in the Big-Dye Terminator cycle sequencing ready reaction kit (Applied Biosystems). Product was sequenced from both strands to confirm their identity with reference sequence.

**Preparing Sequencing Reaction**

DNA template = ………………….…..4 µl

Big Dye=…………………..............1µl

5X Sequencing Buffer=…………... 2 µl

R/F Primer (10pmol/µl)=…………..1µl

Nuclease free water=……………... 2 µl

Total Reaction Volume = 10 µl

PCR conditions for both forward and reverse amplification were 94°C (2 minutes), 35cycles [94°C (30 seconds), 58°C (30seconds) and 72°C (1 minute)], and 72°C (10minutes). PCR products were purified and sequenced on ABI PRISM 3100 sequencer genetic analyzer (described previously). Sequences were analyzed manually by using Chromas software version (v 1.45). Homology studies of the nucleotide sequences of amplified and sequenced P2X4 with known nucleotide sequence which was kindly provided by Dr. Ishtiaq Qadri (Director, NCVI, National University of Science and Technology, Pakistan) was done through standard Basic Local Alignment Search Tool software available at NCBI website. The sequence of P2X4 showed 100% homology with reference sequence.

**2: PCR Amplification of Transfected Null Vector:**

a. 2X fermentas master mixture ……………. 10 µl
b. Forward Primer (10pmol) …………………1 µl
c. Reverse Primer (10pmol) ………………….…1 µl
d. cDNA used (1µg/µl)………………...…… 1 µl
e. Nuclease free water ……………….…….. 7 µl

Total ReactionVolume = 20 µl

PCR conditions were 95°C (3minutes), 30 cycles [95°C (30 seconds), 52°C (30 seconds) and 72°C (45 seconds)], and 72°C (10 minutes). Amplification product was run on 2% agarose gel and visualized with ethidium bromide staining. A 100 or 50-bp ladder (Fermentas) was run as DNA size marker. Amplified product of expected size was obtained.

**B). Western Blot Analysis:**

To study protein expression of P2X4 in stable cell line, 100µg of total protein were loaded in each well on 10% SDS–PAGE gels and electrophoretically blotted onto a nitrocellulose membrane (Bio-Rad). The expression level of P2X4 was determined using antibody specific to P2X4. The protein concentration of cell extracts was determined using a protein assay reagent (Bio-Rad). The membranes were blocked for over –night at 4°C with phosphate –buffered saline containg 5% skim milk. After being washed with 1xPBS containing 0.1% Tween 20, the membranes were incubated with primary antibody specific to P2X4 (Chemicon International, Cat no.AB5226) for over –night at 4°C. Wash the cells three times with 1X PBST and treated with secondary antibody (Chemicon International, Cat no.AP132A) for over –night at 4°C. After being washed with PBST proteins expressions were evaluated using BCIP/ NBT solution.

Western blot and RT-PCR analysis were performed at 48h and 96 h of post transfection and after that continuous selection with G418 for of 1 month. Singles clones were also analyzed at RT-PCR level and through Western blot analysis. Highest null vector (NV) mRNA expressing and P2X4, mRNA and protein expressing clones (transfected with 4µg plasmid DNA) were selected for further studies.

**C). Viral inoculation and sample collection:**

Stable cell lines 293T/P2X4 (stable clone transfected with and stably overexpressing P2X4 protein) and 293T/NV (stably expressing vector alone or null vector) were grown in 60mm petri-dishes to 70% confluency and 40% confluency in two different groups. Required confluency obtained after 2days of subculturing, cells were washed with 1XPBS and 1ml of complete DMEM supplimentd with G418 (500µg/ml) was added in each dish. Cells were inoculated with human HCV sera and control sera (240µl serum was mixed gently in 1ml complete DMEM medium/ per dish). Control sera and sera from chronically infected HCV patients (local population) with high viral titers and genotype 3a were obtained from Diagnostic Research Laboratories of centre (CEMB). Control sera (normal sera) were obtained from subjects negative for Hepatitis A, B and C, CMV and HIV. The viral load in the used HCV sera

was quantified by real time PCR using HCV Real-TM Quant kit (REF, TVI-96/2FRT C SP, Sacace Biotechnologies, Italy) following the manufacturer's instructions. After 40-48h of inoculation, adherent cells were washed two times with IX PBS to get rid of the remaining infection serum. Incubation was continued in complete DMEM containing G418 (500μg/ml). The cells were maintained overnight at 37°C in a humidified environment containing 5% $CO_2$. On day $5^{th}$ RNA was isolated from cells using Gentra RNA Isolation Kit (Puregene, Minneapolis, MN 55441, USA) according to the following protocol.

All the tubes, pipette tips and containers used in the extraction were either certified RNAase free or were made RNAase free by treating with 1% DEPC treated water and were autoclaved before use.

- Removed the media from each petri-dish.
- Washed the cells twice with 1X PBS.
- Dispensed 500 µl of cell lysis solution per petri-dish.
- Added 5µl of HCV internal control (FAM dye) per dish.
- Lysed the cells properly with loop.
- Transferred the mixture to properly labeled appendroff.
- Dispensed 150 µl of protein precipitation solution in it & incubated in ice for 10 minutes.
- Removed from ice & centrifuge for 8minutes at 132000 RPM.
- Added the supernatant into new appendroff tube containing 500 µl of isopropanol.
- Centrifuged for 8miutes at 13200 RPM.
- Discarded the supernatant.
- Dispensed 500 µl of 70% ethanol in the eppendrof tube containing RNA pallet.
- Centrifuged again at 13200 rpm for 5min.
- Discarded supernatant & dried RNA pellet by inverting eppendrof tube over tissue paper 10 minutes inside the hood.
- Dispensed 20-50 µl of RNA hydration water into each eppendrof.
- Placed in at -20c.

- **Preparation of PCR Mix for RT PCR:**

- Added 300 µl of PCR mix-1 & 200 µl of PCR mix-2 into DTT tube (enhancer) & mixed.
- Added in to it 20 µl of Hot start polymerase in it (Taq F polymerase) & 10 µl of M-MLV Revertase (Reverse Transcriptase).Vortexed thoroughly & centrifuge briefly, stored at -4°C.
- **Protocol of RT-PCR:**

- 12.5 µl of RT-PCR mix & 12.5 µl of cellular RNA were dispensed into smart cycler tubes.
- Four standards were prepared for each run, two each of quantitation standards HCV & quantitation standard IC.
- Smart cycler tubes were briefly centrifuged and were run in thermocycler.
- Following parameters were followed for Quantitative real time RT-PCR.

  - Stage 1 Hold $50^{o}C$ – 1800 seconds.
  - Stage 2 Hold $95^{o}C$ – 900 seconds.
  - Stage 3 2- Temperature cycle $95^{o}C$ – 20 seconds, & $60^{o}C$ – 40 seconds.

- **Formula for the calculation of HCV RNA concentration.**

Following formula was used to calculate the concentration of HCV RNA of each sample.

$$\frac{\text{Cy3 STD/Res}}{\text{Fam Std/Res}} \times \text{Coefficient IC} = \text{IU HCV/ml.}$$

Co-efficent, IC = internal control, which is specific for each lot.

Successfully inoculated RNA samples (with significant HVC titers) were quantified and further processed for cDNA synthesis as previously described in Sec.3.1. On day 9$^{th}$, RNA was

isolated, viral titers were quantified through real time PCR. RNA samples with significant viral titers were further processed for cDNA synthesis following the same procedures as described previously. Inoculated cell lines showed significant HCV viral titers on day 5 and day 9 but not on day 14. So, cDNA of four different cell lines P2X4/HCV, P2X4/NR, NV/HCV and NV/NR after day 5 of inoculation and day 9 of inoculation were processed for study III.

# PART- III

**STUDY THE P2X4 RECEPTOR SIGNALING IN THE REGULATION OF ANTIOXIDANTS, INDUCTION OF PROINFLAMMATORY, PROFIBROTIC, VASOACTIVE CYTOKINE AND ADIPOKINES, ECM PROTEINS IN ESTABLISHED CELL CULTURE BASED SYSTEM:**

**A). Designing of PCR primers for different ECM markers:**

Sense and anti-sense primers for the amplification of different ECM (extracellular matrix proteins) markers Laminin, Elastin were designed using primer3 software.

Following gene specific primers were used for PCR amplifications from cDNA.

| S.No | Primer Name | Primer Sequence: 5'-3' Sequence |
|---|---|---|
| 1 | ELAS – F | CAGGAGTTGGTGGCTTAGGA |
| 2 | ELAS- R | CTGGAGCCTTGGGCTTAACT |
| 3 | LAM- F | CACCAAGTCCTGTCACCTGT |
| 4 | LAM- R | CTGGTGTGGAACTTGAGACG |

**Table4: List of Primer sequences used for amplification of ECM markers**

**●Optimization of Laminin:**

a. 10X PCR Buffer ……………………………..... 2μl
b. $MgCl_2$ (25mM) …………………………...…..2μl
c. dNTPs (500 μM) …………………..……....…1μl
d. *Taq*. DNA Polymerase(fermentas) (5U/μl) …..0.4μl(2U)
e. Forward Primer (5pmol/ul) …………………0.3μl
f. Reverse Primer (5pmol/ul) …………………..0.3μl
g. cDNA used (1μg/μl)………………..………1μl
h. Spermine……………………………………..1.25μl
i. Nuclease free water ………….. …………. 11.75μl

Total Volume = 20μl

The samples were preheated at 94°C for 3minutes and then run 35 cycles with the following parameters: at 94°C for 1 minute, 62°C for 45 seconds and 72°C for 1 minute. Final extension was done for 10 minutes at 72°C. PCR product was checked on agarose gel electrophoresis using 2% agarose. The amplified PCR product of expected size (210 bp) was obtained.

**●Optimization of Elastin:**

a. 2X fermentas master mixture ………….……10μl
b. Forward Primer (5pmol/ul) ……………………..1μl
c. Reverse Primer (5pmol/ul) ……………...……….1μl
d. cDNA used (1μg/μl)…………………………...…. 1μl
e. Spermine ………………………………........1.25μl
f. Nuclease free water…………………………....5.75μl

Total Reaction Volume = 20 μl

The samples were preheated at 94°C for 45seconds and then run 35 cycles with the following parameters: at 94°C for 1 minute, 57°C for 45 seconds and 72°C for 1 minute. Final extension was done for 10 minutes at 72°C. PCR product was checked on agarose gel electrophoresis using 2% agarose. The amplified PCR product of expected size (210 bp) was obtained.

**B).Quantitative Real Time PCR.**

Reaction conditions and PCR profile were optimized for real time PCR.

**Optimization of Collagen type IV, Elastin and P2X4 (Transfected)**

**For qRT-PCR:**

a. 2X Maxima™ SYBR Green qPCR Master Mix ……………. 12.5µl
b. Forward Primer (6.4pmol/ul) ……………………………………1.6µl
c. Reverse Primer (6.4pmol/ul) …………………………………...1.6µl
d. cDNA used (1µg/µl)……………………………………………. 1µl
e. Nuclease free water ……………………………………………. 8.3µl

Total Reaction Volume = 25 µl

**Optimization of Laminin for qRT-PCR:**

f. 2X Maxima™ SYBR Green qPCR Master Mix ……………. 12.5µl
g. Forward Primer (2pmol/ul) ……………………………………0.5 µl
h. Reverse Primer (2pmol/ul) …………………………………...0.5 µl
i. cDNA used (1µg/µl)……………………………………………. 1µl
j. Nuclease free water ……………………………………………. 10.5µl

Total Reaction Volume = 25 µl

Real time PCR reactions were run on Cepheid smart cycler II (France) at same PCR profiles which were used to optimized PCR products at conventional PCR (ABI-2700) thermal cycler).

**C). Cu/Zn-SOD, HO-1, TNF-α, TGF-β1, Leptin, Angiotensin II, Elastin, Laminin and P2X4 (Transfected) gene expression analysis:**

The expression of Cu/Zn-SOD, HO-1, TNF-α, TGF-β, Leptin, Angiotensin II, Elastin, Laminin and P2X4 (Transfected) genes was analyzed in cell lines 293T/P2X4-HCV, 293T/P2X4-NR, 293T/NV-HCV and 293T/NV-NR (after day 5 of inoculation and day 9 of inoculation).

The gene expression analysis was done using real time Real Time PCR as already described.

**D). Statistical Analysis:**

Statistical analysis was performed with GraphPad Prism 5 software Version 5.02. All data were presented as mean ± SEM. Comparisons between parameters were performed using ANOVA followed by Bonferroni or unpaired t test. A *p*-value of less than or equal to 0.05 was considered statistically significant.
